# METTL3 Promotes Lipid Deposition and Pulmonary Fibrosis by Destabilizing PLIN2 in a m6A‐Dependent Manner

**DOI:** 10.1111/jcmm.71239

**Published:** 2026-09-09

**Authors:** Qiping Liu, Rui Xu, Xianzhi Du

**Affiliations:** ^1^ Department of Respiratory and Critical Care Medicine Second Affiliated Hospital of Chongqing Medical University Chongqing China

**Keywords:** idiopathic pulmonary fibrosis, lipid, m6A methylation, METTL3, PLIN2

## Abstract

Idiopathic pulmonary fibrosis (IPF) is a lethal lung disorder that is associated with aberrant lipid metabolism. N6‐methyladenosine (m6A) methylation is involved in IPF progression. However, whether METTL3, a m6A methyltransferase, modulates lipid metabolism in IPF remains unknown. In this study, we aimed to investigate the effects of METTL3 on fibrosis and lipid accumulation and identify the molecular mechanism. PF mouse model was generated by bleomycin. WI‐38 cells were stimulated with TGF‐β1 to mimic the IPF‐caused damage. Lipids were observed using Nile Red staining and marker detection. Fibrosis was evaluated using Masson's Trichrome staining and marker detection. The molecular mechanism was analysed by methylated RNA immunoprecipitation and dual‐luciferase reporter assay. We found that METTL3 is highly expressed in IPF mice and TGF‐β1‐induced WI‐38 cells. Knockdown of METTL3 inhibited lipid accumulation and fibrosis In Vitro and In Vivo. Additionally, interference with METTL3 suppressed the m6A methylation of PLIN2, enhanced its stability, and increased its expression. Knockdown of PLIN2 reversed the inhibition of lipid accumulation and fibrosis caused by METTL3 silence. In conclusion, interfering with METTL3 attenuates IPF progression by suppressing lipid deposition, which is associated with the increased stability of PLIN2 suggesting a promising therapeutic target for IPF.

## 1 Introduction

Idiopathic pulmonary fibrosis (IPF) is a chronic progressive and ultimately lethal lung disorder of unknown aetiology characterized by the accumulation of extracellular matrix (ECM) and the destruction of normal lung architecture [1, 2]. It has been reported that the annual incidence of IPF is roughly 10 per 100,000 individuals [3] and the median survival time is only 3 years [4]. It is an age‐related lung disease. As the aging population continues to progress, the burden of IPF is expected to steadily increase [5]. Antifibrotic therapy is an effective strategy to decelerate disease progression but complications can worsen disease symptoms or promote death [6]. Therefore it is necessary to clarify the pathogenesis of IPF. Nevertheless the pathogenesis of IPF is complex and multifactorial involving genetic predispositions environmental exposures, and dysregulated cellular processes [7]. Among these lipid metabolism has emerged as a critical factor in the pathogenesis of IPF [8, 9]. Lipids are not only structural components of cellular membranes but also serve as signalling molecules that modulate cellular functions including fibroblast activation and ECM production. Thus, elucidating the regulatory mechanism of lipid metabolism during IPF is helpful to provide possible strategies for the treatment of IPF from the perspective of lipid change.

The N6‐methyladenosine (m6A) modification is the most prevalent and well‐studied RNA modification playing a pivotal role in the regulation of gene expression and human health [10]. It has been implicated in various diseases such as cancer cardiovascular disorders and fibrotic diseases [11, 12, 13, 14]. Importantly m6A is involved in IPF. It may be a diagnostic and prognostic marker as well as a novel therapeutic target [15, 16]. Methyltransferase like 3 (METTL3) which is a key component of the m6A methyltransferase complex, is clarified to catalyse m6A methylation that mediates the addition of a methyl group to the N6 position of adenosine residues in RNA primarily in mRNAs [17]. It has been demonstrated that silencing of METTL3 inhibits the conversion of fibroblasts into myofibroblasts and thus suppresses fibrosis progression [18]. However whether METTL3 regulates lipid metabolism in IPF remains largely unknown.

Perilipin 2 (PLIN2) a member of the perilipin family regulates lipid accumulation and utilization. Most cells have lipid droplets with PLIN2 on the surface and PLIN2 controls the entry of lipases and their co‐factors into substrate lipids in the lipid droplets to regulate lipolysis [19]. PLIN2 has been implicated in various diseases involving lipid dysregulation such as myocardial infarction obesity and fatty liver [20, 21]. Moreover PLIN2 is closely linked to lipogenic differentiation in lung fibrosis [22]. Nevertheless its potential interplay with METTL3 has not been well explored.

In this study we aimed to elucidate the impact of METTL3 and its downstream target PLIN2 on lipid metabolism and fibrogenesis in IPF. Moreover we identified how METTL3 regulated PLIN2. Our findings may provide novel insights into the pathogenesis of IPF and open up new therapeutic targets for IPF.

## 2 Materials and Methods

### 2.1 IPF Mouse Model

C57BL/6 mice (male and female; age: 10‐week‐old; weight: 22–25 g) were purchased from Cavens (Changzhou, China). These mice were kept at 23 ~ 25°C with 12/12 h light/cycle conditions with free access to water and food. All mice were randomly divided into four groups, including control (CON) bleomycin (BLM) BLM + short hairpin RNA negative control (shNC) and BLM + short hairpin RNA METTL3 (shMETTL3) with 20 mice in each group. To induce the BLM model mice were anaesthetised by inhaling isoflurane. Then the mice were intratracheally administered 50 μL normal saline containing 1.8 U/kg BLM (Sigma‐Aldrich St. Louis MO USA). The CON group mice were given equal amounts of normal saline in the same way. To evaluate the role of METTL3 adenovirus carrying shNC or shMETTL3 was diluted in normal saline and 5 × 10^8^ PFU/mouse adenovirus was intratracheally administered after BLM administration. The survival rate was observed until 28 days. At 28 days, all the surviving mice were euthanatized by isoflurane inhalation. The lung tissues were obtained. The experimental data from each group of 6 mice were statistically analysed.

### 2.2 Histopathological Analysis

The lungs were fixed with 4% paraformaldehyde for 24 h. The tissues were dehydrated permeabilized embedded and cut into paraffin sections with a thickness of 5 μm. Then the histopathology was analysed using the haematoxylin and eosin (H&E) stain kit (Sorlarbio, Beijing, China). Collagen fibres were observed using the Masson's Trichrome stain kit (Sorlaibio). The procedures were performed according to the manufacturer's protocols.

### 2.3 Nile Red Staining Assay

Lipids in lung tissues were observed using the lipid fluorescent staining kit (Nile Red Method) (Solarbio). Briefly the tissues were embedded in optimal cutting temperature (OCT) compound and cut into 10 μm thick frozen sections. The sections were fixed using the lipid fixative for 20 min and washed with phosphate‐buffered saline (PBS). Next the sections were incubated with Nile Red staining working solution for 10 min in the dark. 4′,6‐diamidino‐2‐phenylindole (DAPI; Sigma‐Aldrich) was used to stain the cell nucleus. After washing with PBS, the fluorescence was observed using fluorescence microscopy.

### 2.4 Immunohistochemistry

The paraffin sections were subjected to antigen retrieval using 0.01 M sodium citrate buffer under boiling conditions. Next, 3% hydrogen peroxide was added to inhibit the endogenous catalase activity. Each section was incubated with primary antibody against PLIN2 (sc‐377,429, 1:500; Santa Cruz Biotechnology Santa Cruz, CA, USA) at 4°C overnight, and incubated with secondary antibody HRP‐conjugated anti‐mouse IgG (sc‐525,409, 1:100; Santa Cruz Biotechnology) at 37°C for 30 min. After sealing, the sections were viewed under a microplate.

### 2.5 Cell Culture

Human embryo lung cells (WI‐38) were obtained from Procell (Wuhan China). These cells were maintained in Dulbecco's modified Eagle's medium (Gibco) containing 10% fetal bovine serum (FBS; Gibco) and 1% penicillin/streptomycin (Gibco Grand Island NY USA) at 37°C with 5% CO_2_.

To generate injured cells In Vitro to stimulate IPF‐damaged cells WI‐38 cells were exposed to 10 ng/mL human recombinant TGF‐β1 (Sigma‐Aldrich) for 48 h.

### 2.6 Cell Transfection

Short hairpin (sh) RNA targeting METTL3 (shMETTL3) shPLIN2 and sh negative control (shNC) were acquired from Ribobio (Guangzhou, China). WI‐38 cells were seeded into six‐well plates and transfected with these shRNA plasmids using Lipofectamine 2000 (Invitrogen, Carlsbad, CA, USA) for 48 h.

### 2.7 Quantitative Real‐Time PCR (qRT‐PCR)

Total RNA was extracted using TRIzol reagent (Invitrogen), and RNA concentration was measured by spectrophotometry at A260/A280. cDNA first chain was generated from 1 μg of RNA using HiScript III RT SuperMix for qPCR (Vazyme, Nanjing, China). RNA expression was measured using the ChamQ SYBR qPCR Master Mix (Vazyme) on a CFX96 real‐time PCR system. The relative expression was calculated using the 2^−ΔΔCt^ method with the normalization of β‐actin. The primary sequences were as follows: Col‐1 forward 5′‐GATTCCCTGGACCTAAAGGTGC‐3′, Reverse 5′‐AGCCTCTCCATCTTTGCCAGCA‐3′; α‐SMA Forward 5′‐CTATGCCTCTGGACGCACAACT‐3′, Reverse 5′‐CAGATCCAGACGCATGATGGCA‐3′; METTL3 Forward 5′‐CTATCTCCTGGCACTCGCAAGA‐3′, Reverse 5′‐GCTTGAACCGTGCAACCACATC‐3′; FAS Forward 5′‐GGACCCAGAATACCAAGTGCAG‐3′, Reverse 5′‐GTTGCTGGTGAGTGTGCATTCC‐3′; ACC1 Forward 5′‐TTCACTCCACCTTGTCAGCGGA‐3′, Reverse 5′‐GTCAGAGAAGCAGCCCATCACT‐3′; SREBP1 Forward 5′‐ACTTCTGGAGGCATCGCAAGCA‐3′, Reverse 5′‐AGGTTCCAGAGGAGGCTACAAG‐3′; PPARα Forward 5′‐TCGGCGAGGATAGTTCTGGAAG‐3′, Reverse 5′‐GACCACAGGATAAGTCACCGAG‐3′; PLIN2 Forward 5′‐GATGGCAGAGAACGGTGTGAAG‐3′, Reverse 5′‐CAGGCATAGGTATTGGCAACTGC‐3′; β‐actin Forward 5′‐CACCATTGGCAATGAGCGGTTC‐3′, Reverse 5′‐AGGTCTTTGCGGATGTCCACGT‐3′.

### 2.8 Western Blotting

Lung tissues or WI‐38 cells were lysed using the RIPA lysis buffer supplemented with protease inhibitor cocktail on ice. Then the samples were centrifuged, and protein concentration was measured in the supernatant using a bicinchoninic acid (BCA) kit (Sigma‐Aldrich). Proteins were separated using 10% sodium dodecyl sulphate‐polyacrylamide gel electrophoresis, followed by transfer onto polyvinylidene fluoride membranes. Following blocking using 5% skimmed milk in Tris‐buffered saline‐Tween 20 (TBST) the membranes were incubated with primary antibodies overnight at 4°C, and then incubated with secondary antibody for 1 h at room temperature. TBST was used to wash the membranes after each incubation. Proteins were detected using the SuperPico ECL Master Mix (Vazyme) and imaged.

The primary antibodies used here include anti‐Col‐1 (sc‐59,772, 1:1000) anti‐α‐SMA (sc‐130,617, 1:1000) and anti‐β‐actin (sc‐8432, 1:500). The secondary antibody was HRP‐conjugated anti‐mouse IgG (sc‐525,409, 1:1000). All antibodies were acquired from Santa Cruz Biotechnology.

### 2.9 Immunofluorescence

WI‐38 cells were grown on the glass slide to prepare cell climbing slices. The clices were fixed with 4% paraformaldehyde for 20 min and subsequently incubated with 0.2% Triton X‐100 for 15 min. Block was performed using 5% bovine serum albumin (Sigma‐Aldrich). The cells were incubated with anti‐α‐SMA (sc‐130,617, 1:200; Santa Cruz Biotechnology) overnight at 4°C, and incubated with CruzFluor 488‐conjugated anti‐mouse IgG (sc‐533,653, 1:100; Santa Cruz Biotechnology) at 37°C for 2 h. DAPI was added to incubate the cells in the dark for 15 min. The fluorescence was observed by fluorescence microscopy.

### 2.10 Oil Red O Staining Assay

Lipid droplets were observed using the Oil red O staining kit (Sbjbio Nanjing China). Briefly WI‐38 cells were washed twice with PBS and fixed with ORO stationary liquid for 20 min. The cells were then stained with Oil red O solution for 15 min at room temperature. After washing slightly with 60% isopropanol to remove the dye solution the cells were observed under a light microscope.

### 2.11 Methylated RNA Immunoprecipitation (MeRIP)

The m6A levels of PLIN2 were measured using the EpiQuik MeRIP kit (Epigentek Farmingdale NY USA). Fragmented total RNA was incubated with magnetic beads A/G precoated with 5 μg antibodies targeting m6A and IgG at 4°C for 2 h. After eluting the beads PLIN2 expression was detected using qRT‐PCR.

### 2.12 RNA Immunoprecipitation (RIP)

Magna RIP RNA‐binding protein immunoprecipitation kit (Merck Millipore Billerica MA USA) was used to evaluate the interaction between METTL3 and PLIN2. Protein A + G magnetic beads were pre‐coated with 5 μg antibodies targeting METTL3 and IgG, followed by incubation with cell lysates at 4°C overnight. After washing RNA was purified and PLIN2 expression was detected using qRT‐PCR.

### 2.13 M6A Sites Prediction

The sites that could be modified by m6A were predicted using the SRAMP database.

### 2.14 Dual‐Luciferase Reporter Assay

The wild‐type (WT) sequences of PLIN2 were inserted into pGL3 vectors (Promega, Madison, WI, USA). Meantime, three possible m6A sites in the PLIN2 sequences were mutated (MUT) and also inserted into the same vectors. The cells were co‐transfected with WT or MUT reporter plasmids, shNC or shMETTL3 and pRL‐TK vector (Promega) using Lipofectamine 2000 for 48 h. The firefly and *Renilla* luciferase activities were measured using the dual‐luciferase reporter assay system (Promega).

### 2.15 RNA Stability Detection

WI‐38 cells were exposed to 5 μg/mL actinomycin D (Sigma‐Aldrich) for 0, 4, 8, and 12 h. Total RNA was isolated for PLIN2 expression detection.

### 2.16 Statistical Analysis

Statistical analysis was performed using the GraphPad Prism 8.0 software. Differences were analysed using the Student's *t*‐test (two groups) and one‐way or two‐way ANOVA (more than two groups). Results were represented as means ± standard deviation. Statistically significant data were defined as *p* < 0.05.

## 3 Results

### 3.1 METTL3 Is Highly Expressed in BLM‐Induced Mouse and TGF‐β1‐Induced WI‐38 Cells

**FIGURE 1:**
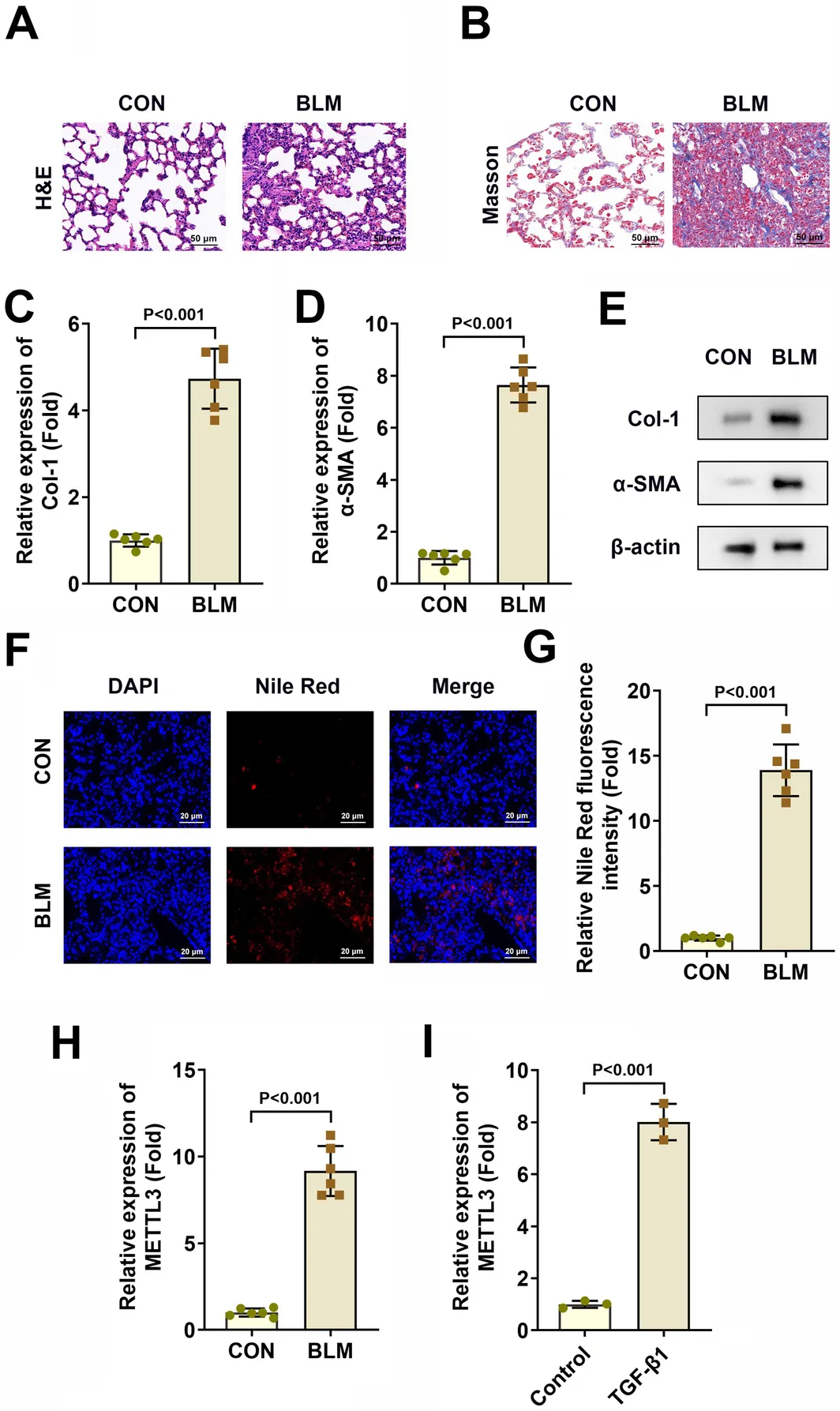
METTL3 is highly expressed in BLM‐induced mouse and TGF‐β1‐induced WI‐38 cells. Represent images of (A) H&E and (B) Masson's trichrome staining of the lung paraffin sections. Both scale bar = 50 μm. (C) Col‐1 and (D) α‐SMA expression was detected using qRT‐PCR. (E) Protein levels of Col‐1 and α‐SMA were measured using western blotting. (F) Represent images of Nile red staining of the lung frozen sections. DAPI was used to stain the nucleus. Scale bar = 20 μm. (G) Quantification results of Nile red staining. (H) METTL3 expression in the lungs of mice in each group was measured using qRT‐PCR. (I) METTL3 expression in the WI‐38 cells treated with TGF‐β1 or not was detected using qRT‐PCR. *n* = 6 for animal experiments. *n* = 3 for In Vitro experiments.

To analyse the pathogenesis of IPF, we used BLM to generate a mouse model. H&E and Masson's trichrome staining were performed. The results showed that BLM destructed the lung structure of mice (Figure 1A) and promoted collagen accumulation and deposits (Figure 1B). Next, the levels of fibrosis markers were measured using qRT‐PCR and western blotting. As shown in Figure 1C–E, Col‐1 and α‐SMA levels were upregulated in the lung tissues of mice. Aberrant lipid metabolism is associated with IPF. Therefore, Nile red staining was conducted to measure lipid content. We found that BLM treatment increased lipid deposition in the lung tissues (Figure 1F,G). In this study, we sought to investigate the role of METTL3 in IPF. Thus, its expression was detected in the lung and the results showed that METTL3 expression was upregulated in BLM‐induced mice (Figure 1H). Additionally, we cultured WI‐38 cells In Vitro and treated them with TGF‐β1 to mimic IPF environment. It could be seen that METTL3 expression was higher in the TGF‐β1‐stimulated WI‐38 cells than that in the cells of the control group. (Figure 1I). The results demonstrated that IPF is associated with lipid accumulation in the lungs, accompanied by the increased expression of METTL3. We hypothesized that METTL3 may regulate lipid metabolism in IPF.

### 3.2 Knockdown of METTL3 Suppresses Lipogenesis and Fibrosis

**FIGURE 2:**
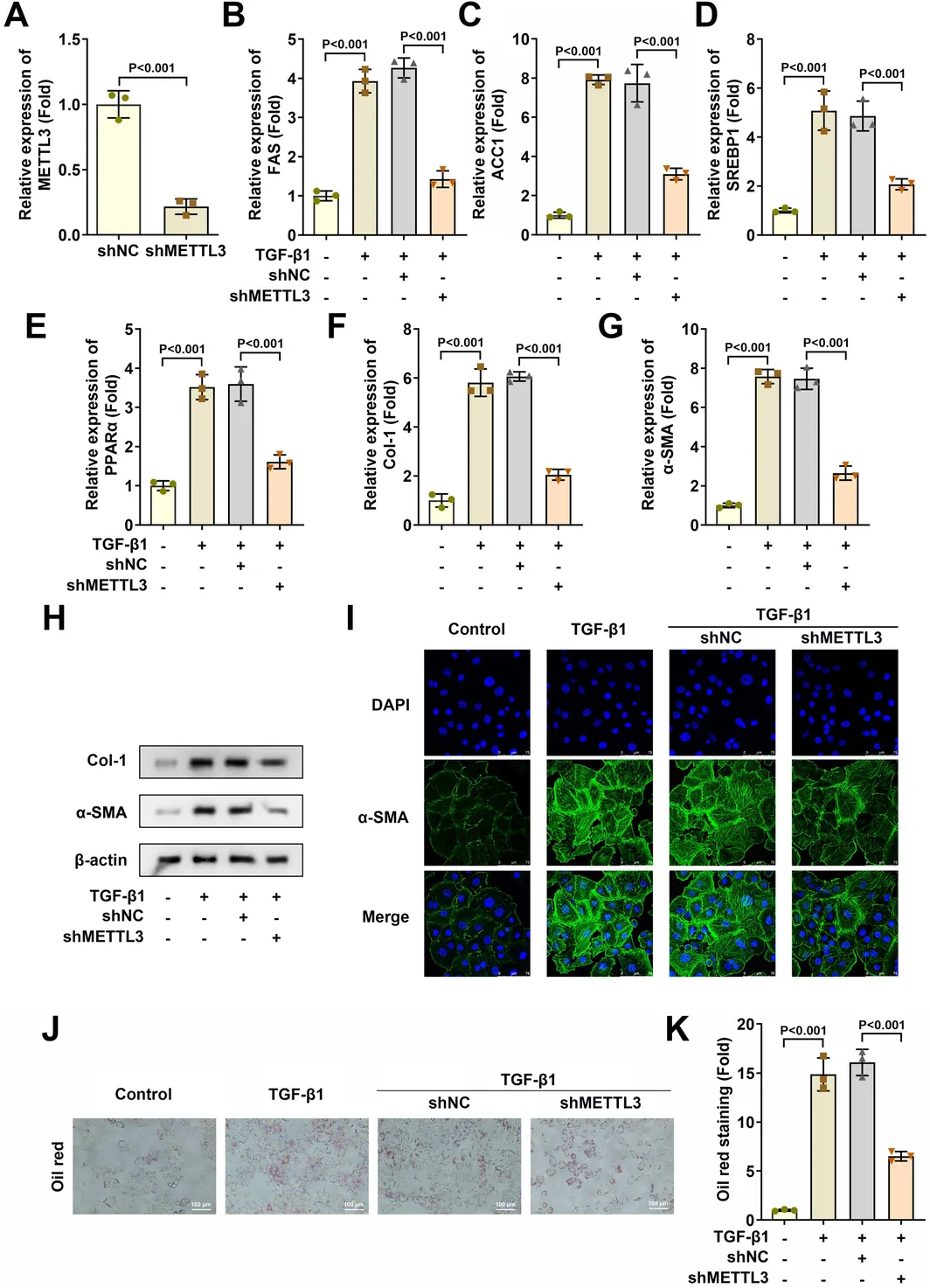
Knockdown of METTL3 suppresses fibrosis and lipid deposits. (A) METTL3 expression in WI‐38 cells after shNC and shMETTL3 transfection. qRT‐PCR was used to detect the expression of (B) FAS, (C) ACC1, (D) SREBP1, and (E) PPARα. (F) Col‐1 and (G) α‐SMA expression was detected using qRT‐PCR. (H) Protein levels of Col‐1 and α‐SMA were measured using western blotting. (I) α‐SMA levels were examined using immunofluorescence. Scale bar = 75 μm. (J) Lipid droplets were observed by Oil red O staining assay. Scale bar = 100 μm. (K) Quantification results of Oil red O staining. All *n* = 3.

Subsequently, the effect of METTL3 on WI‐38 cellular behaviours was explored. METTL3 expression was downregulated after transfecting its shRNA sequences, compared with shNC (Figure 2A). The expression of lipogenesis markers, including FAS, ACC1, SREBP1, and PPARα, was detected using qRT‐PCR. We observed that TGF‐β1 elevated their expression in WI‐38 cells, which was counteracted by METTL3 knockdown (Figure 2B–E). The fibrosis markers, Col‐1 and α‐SMA, whose expression was measured in each group of WI‐38 cells. The results showed that both Col‐1 and α‐SMA levels were increased in TGF‐β1‐induced WI‐38 cells, compared with non‐treated cells. In addition, their levels were decreased in the METTL3‐knocked down cells, compared with its negative control group. (Figure 2F–H). Besides, immunofluorescence was also conducted to measure α‐SMA expression, and the results were the same as mentioned above. (Figure 2I). Oil red O staining results revealed that TGF‐β1 induced the increase of lipid droplets, which was partly abolished by METTL3 knockdown. (Figure 2J,K). These results indicated that TGF‐β1‐treated WI‐38 cells showed severe fibrosis and lipogenesis, and METTL3 knockdown significantly improved fibrosis and lipogenesis.

### 3.3 Interfering With METTL3 Stabilizes PLIN2 via Suppressing the m6A Modification

**FIGURE 3:**
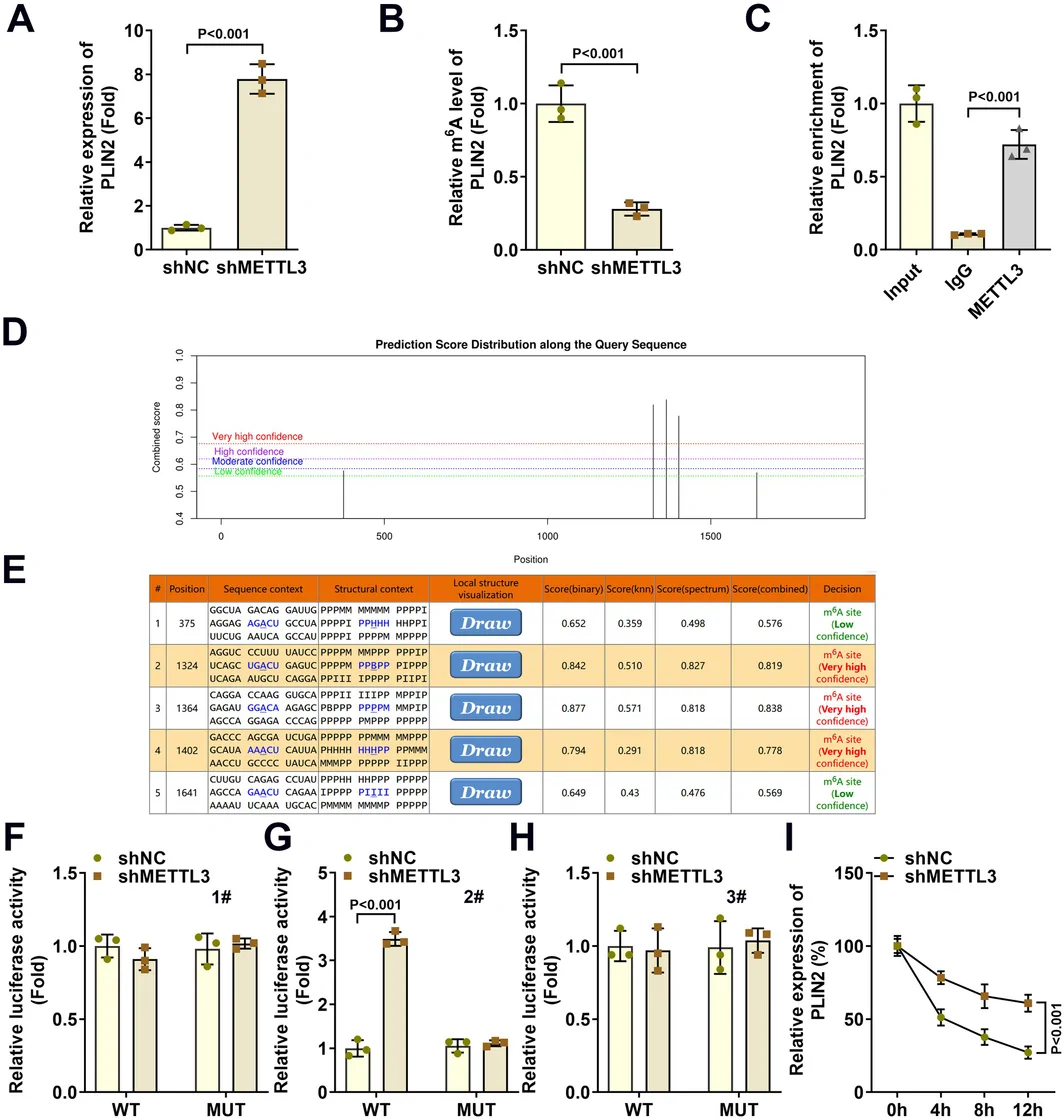
Interfering with METTL3 stabilizes PLIN2 via suppressing the m6A modification. (A) After METTL3 was knocked down, PLIN2 expression was detected using qRT‐PCR. (B) The m6A levels of PLIN2 were measured after knocking down METTL3 using MeRIP. (C) The interaction between METTL3 and PLIN2 was evaluated using RIP. (D, E) Potential m6A sites in PLIN2. (F‐H) The m6A sites were verified using dual‐luciferase reporter assay. (I) PLIN2 stability after METTL3 knockdown. All *n* = 3.

PLIN2 is a key driver of lipid accumulation [23]. Notably, a previous study has reported that PLIN2 is differentially expressed in fibroblasts in pulmonary fibrosis [24]. Since we discovered the regulatory effect of METTL3 on lipid metabolism, in order to explore the molecular mechanism of METTL3, we studied its regulatory effect on PLIN2. We first measured PLIN2 expression and found that silencing of METTL3 caused the upregulation of PLIN2. (Figure 3A). Later, based on the catalytic effect of METTL3 on m6A methylation, we evaluated m6A modification of PLIN2. The results showed that knockdown of METTL3 decreased m6A levels of PLIN2 (Figure 3B). METTL3 was also found to interact with PLIN2 (Figure 3C). Moreover, the m6A sites in PLIN2 were identified. Five potential sites were predicted (Figure 3D,E). Three m6A sites with very high confidence were chosen, named 1#, 2#, and 3#, and a dual‐luciferase reporter assay was performed to confirm the modifying sites. The results showed that silencing of METTL3 increased relative luciferase activity when co‐transfected with WT sequences containing 2#, but did not affect the luciferase activity in the WT 1# and 3# groups (Figure 3F–H). Finally, PLIN2 stability was evaluated, and the results showed that knockdown of METTL3 enhanced PLIN2 stability (Figure 3I). The results showed that interference with METTL3 promotes PLIN2 stability by inhibiting its m6A methylation.

### 3.4 METTL3 Regulates Fibrosis and Lipogenesis by Regulating PLIN2

**FIGURE 4:**
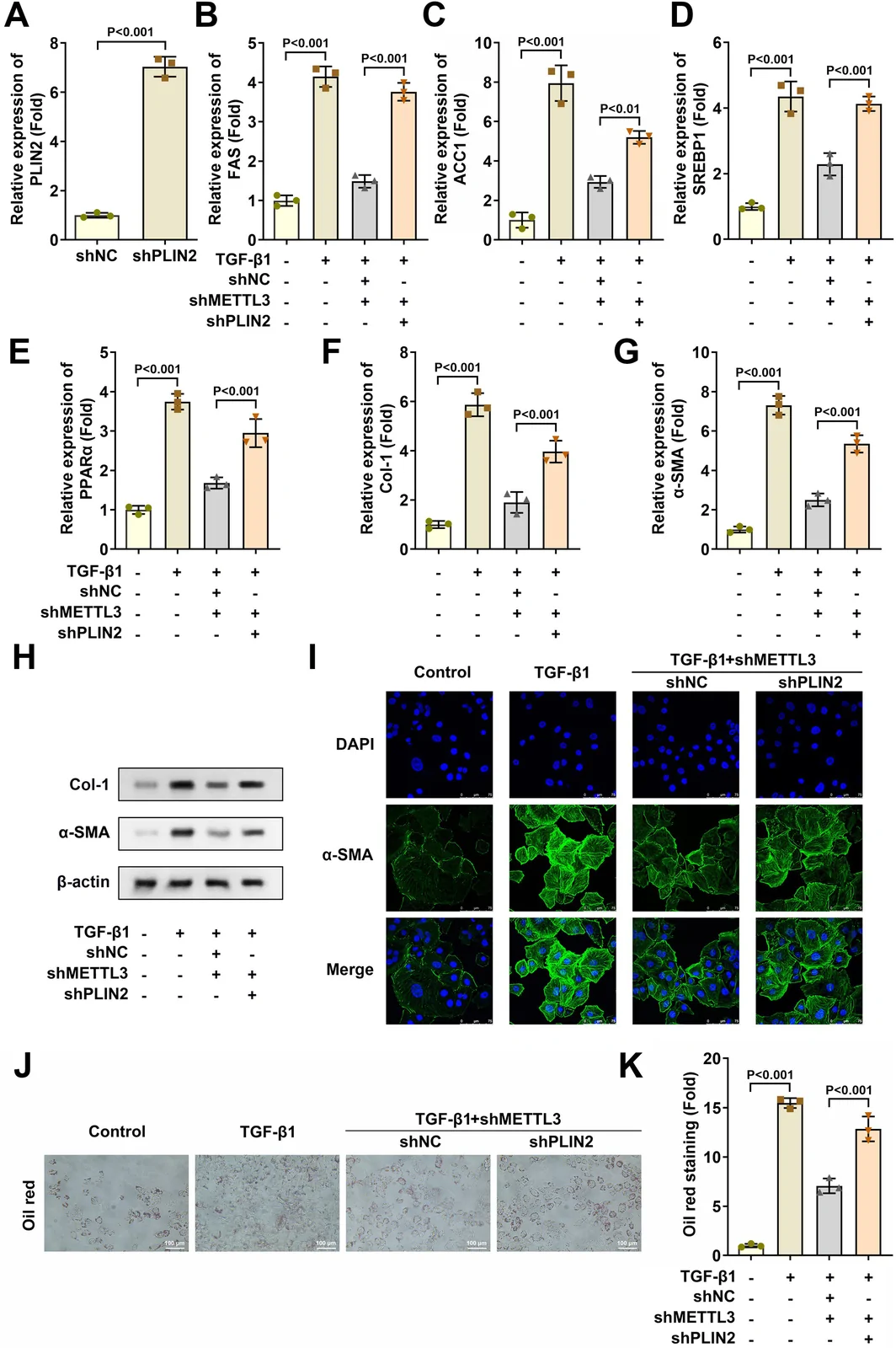
METTL3 regulates fibrosis and lipid metabolism by regulating PLIN2. (A) PLIN2 expression in WI‐38 cells after shNC and sh PLIN2 transfection. qRT‐PCR was used to detect the expression of (B) FAS, (C) ACC1, (D) SREBP1, and (E) PPARα. (F) Col‐1 and (G) α‐SMA expression was detected using qRT‐PCR. (H) Protein levels of Col‐1 and α‐SMA were measured using western blotting. (I) α‐SMA levels were examined using immunofluorescence. Scale bar = 75 μm. (J) Lipid droplets were observed by Oil red O staining assay. Scale bar = 100 μm. (K) Quantification results of Oil red O staining. All *n* = 3.

To Investigate the role impacts of PLIN2 on fibrosis and lipids PLIN2 expression was downregulated after shPLIN2 transfection (Figure 4A). The reduced expression levels of FAS ACC1 SREBP1 & PPARα caused by METTL3 knockdown in TGF‐β1‐induced WI‐38 cells were abrogated by silencing of PLIN2. (Figure 4B–E). In addition Col‐1 and α‐SMA levels in TGF‐β1‐induced cells were decreased after METTL3 knockdown which was reversed by PLIN2. knockdown (Figure 4F–I). The decrease of lipid droplets in TGF‐β1‐Induced cells caused by METTL3 knockdown was counteracted by silencing of PLIN2. (Figure 4J,K). To sum up silencing of METTL3 inhibits fibrosis and lipid deposits by elevating PLIN2 expression.

### 3.5 Silencing of METTL3 Attenuates Fibrosis and Lipid Accumulation in BLM‐Induced Mice

**FIGURE 5:**
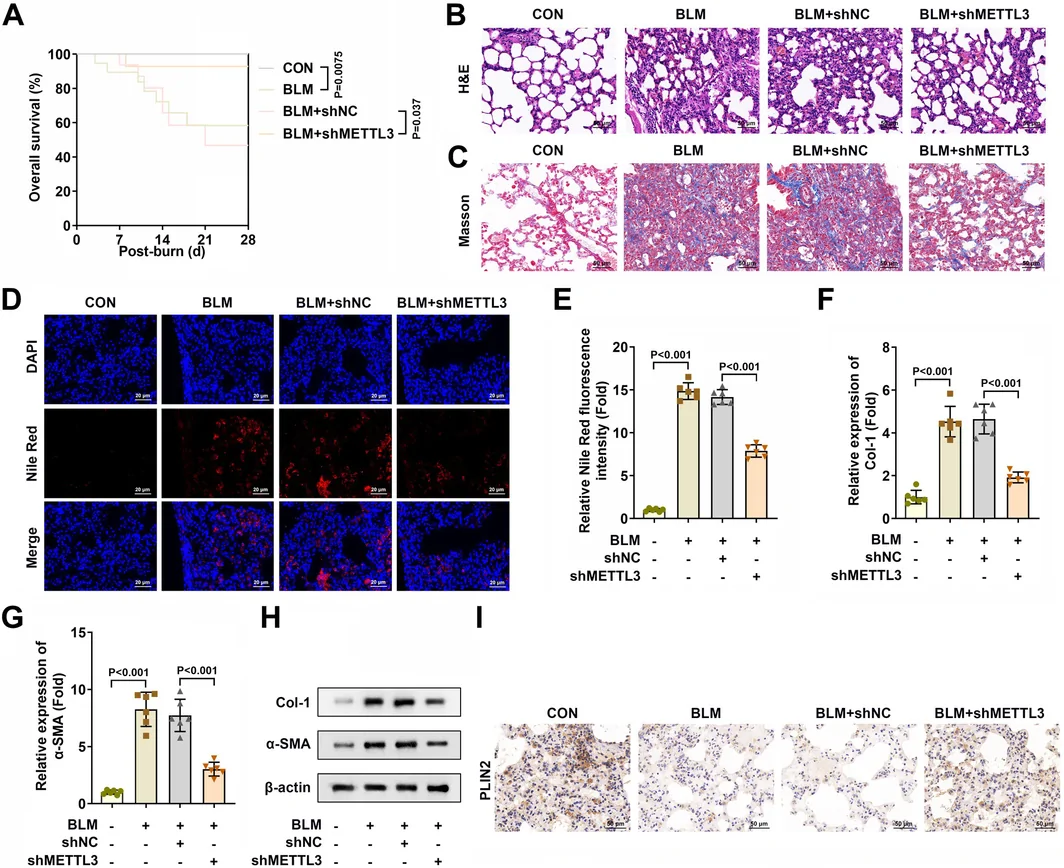
Silencing of METTL3 attenuates fibrosis and lipid accumulation in BLM‐induced mice. (A) Kaplan–Meier curves for survival rate of mice in each group. *n* = 20. Represent images of (B) H&E and (C) Masson's trichrome staining of the lung paraffin sections. Both scale bar = 50 μm. *n* = 6. (D) Represent images of Nile red staining of the lung frozen sections. DAPI was used to stain the nucleus. Scale bar = 20 μm. *n* = 6. (E) Quantification results of Nile red staining. *n* = 6. (F, G) Col‐1 and α‐SMA mRNA expression and (H) their protein levels were detected using qRT‐PCR and western blotting, respectively. *n* = 6. (I) PLIN2 expression in the lungs of mice in each group was measured using immunohistochemistry. Scale bar = 50 μm. *n* = 6.

Based on the role of METTL3 In Vitro, We knocked down METTL3 in mice to explore its role In Vivo. As shown in Figure 5A, BLM caused a decrease in the survival rate of mice, while knockdown of METTL3 improved the survival of BLM‐induced mice. The images of H&E staining and Masson's trichrome staining showed that silencing of METTL3 impeded lung structure destruction and collagen fibre deposition in mice (Figure 5B,C). Lipid accumulation in the lungs was facilitated by BLM, which was counteracted by METTL3 knockdown (Figure 5D,E). The increased expression of Col‐1 and α‐SMA in BLM‐treated mice was decreased after interfering with METTL3 (Figure 5F–H). Besides, the levels of PLIN2, measured using immunohistochemistry, were observed to be downregulated in the BLM‐induced mouse model, whereas METTL3 knockdown reversed this downregulation (Figure 5I). Together, interfering with METTL3 decelerating IPF progression by increasing PLIN2 expression.

## 4 Discussion

In the present study, we found that METTL3, a key m6A methyltransferase, plays a crucial role in the regulation of lipid accumulation and fibrogenesis in IPF. Moreover, PLIN2 was identified as the m6A‐modifying substrate in these processes.

M6A methylation plays a critical role in regulating IPF progression, which is mainly modulated by m6A‐related enzymes. For example, YTHDF1 and METTL3 facilitate the fibroblast‐to‐myofibroblast transition in IPF [18, 25]. Besides, overexpression of YTHDC1 contributes to alleviating lung senescence [26]. In addition, METTL3 is a key regulator of fibrotic diseases, such as cardiac, renal, & liver fibrosis. Specifically, Tu et al. [27] have revealed that METTL3 knockdown inhibits the proliferation and migration of fibroblasts and thereby attenuates cardiac fibrosis. Jung et al. [28] have reported that downregulation of METTL3 ameliorates kidney fibrosis by reducing the expression of fibrotic markers. Shu et al. [29] have found that METTL3 promotes pyroptosis of macrophages to aggravate liver fibrosis. These studies suggest that METTL3 can be involved in fibrosis progression by regulating multiple cellular behaviours in different cells. Fibroblasts have been implicated in the progression of IPF [9]. Moreover, lipid metabolism is considered to be an important pathogenesis of IPF. Thus, we investigated the impacts of METTL3 on lipid metabolism and IPF progression. The results of our study showed that METTL3 expression was increased in the IPF mouse model and TGF‐β1‐induced WI‐38 cells. Knockdown of METTL3 decreased the levels of lipogenesis‐related markers and lipid droplets In Vitro, and inhibited lipid deposition In Vivo. Moreover, we found that silencing of METTL3 decreased the expression of fibrotic markers and reduced collagen fibrosis in the lungs. These findings suggest that METTL3 attenuates IPF progression by suppressing lipid accumulation. Our study presents a new perspective on the impact of METTL3 on IPF progression.

The mechanistic exploration revealed that knockdown of METTL3 inhibited the m6A methylation of PLIN2, thereby enhancing its stability. The finding is in line with the canonical function of m6A modification in regulating mRNA stability. Our results extend the understanding of m6A biology by identifying PLIN2 as a novel target of METTL3. PLIN2 is widely studied in several human diseases. Lipid mediators and pathways in IPF are thought to promote myofibroblast activation, lung remodelling, and fibrosis [30, 31]. Moreover, PLIN2 is commonly dysregulated in age‐related diseases including IPF and affects the pathology and indicates the severity of these diseases [32]. PLIN2 is an intracellular lipid load marker that is associated with lipid formation, size, and degradation [33, 34, 35]. Moreover, PLIN2 is involved in fibrosis. For example, PLIN2 inhibits islet stellate cell activation and attenuates islet fibrosis [36]. Additionally, knockout of PLIN2 in the liver alleviates hepatic steatosis and fibrosis [37]. Herein, the role of PLIN2 in IPF was investigated. We focused on its impacts on lipid accumulation and fibrosis. We demonstrated that knockdown of PLIN2 reversed the effects of METTL3 knockdown on lipid accumulation and fibrosis, which underscores the importance of PLIN2 as a downstream effector of METTL3 in this process. Thus, targeting the METTL3‐PLIN2 axis suggests new therapeutic opportunities for IPF. Increasing PLIN2 expression directly or by silencing of METTL3 can inhibit lipid deposition and fibrosis. However, the role of PLIN2 In Vivo needs to be further elucidated. Future studies should also evaluate the efficacy of targeting PLIN2 in preclinical IPF models.

Although no previous report links METTL3‐m6A‐PLIN2 to IPF, other METTL3/m6A mechanisms in pulmonary fibrosis have been described. Lu et al. [38] have reported that METTL3‐mediated m6A methylation induces fibroblast differentiation into myofibroblasts via the miR‐21/PTEN pathway. Qi et al. [39] have indicated that METTL3‐mediated m6A methylation of circ_0044226 inhibits ferroptosis to attenuate pulmonary fibrosis Via suppressing ferroptosis. Our results further demonstrated the improvement effect of METTL3 knockdown on pulmonary fibrosis. Moreover, PLIN2 knockdown declines mitochondrial function in human dermal fibroblasts in a non‐IPF setting [40]. Given that IPF is age‐related and the dysregulation of PLIN2 in IPF, PLIN2 may interact with METTL3‐dependent m6A modification to affect the fibrosis process, a possibility that warrants further investigation.

Although WI‐38 cells have distinct advantages, such as genetic stability, multiple passage times, ease of handling, high reproducibility of results, and are frequently used to study fibroblast‐to‐myofibroblast transformation in IPF [41]. We also acknowledge their notable limitations. For instance WI‐38 cells cannot fully recapitulate the pathological characteristics of primary lung fibroblasts derived from IPF patients. Moreover embryonic fibroblasts may differ from adult disease‐related fibroblasts in terms of epigenetic and lipid metabolism profiles. Therefore it is necessary to future validate our key findings using other cell models such as MRC‐5 human lung fibroblasts or primary lung fibroblasts from IPF patients. Additionally although we predicted potential m6A sites using SRAMP and validated one candidate (site 2#) by dual‐luciferase reporter assay. we did not perform MeRIP‐seq or RNA‐seq to globally map m6A peaks on PLIN2 transcripts. Future studies employing high‐throughput approaches such as MeRIP‐seq or miCLIP, are warranted to precisely define all m6A sites in PLIN2.

In conclusion our study unveils a novel role of METTL3 in the regulation of lipid metabolism in IPF with its regulation in PLIN2. We demonstrated that silencing of METTL3 inhibits lipid accumulation and fibrosis resolution by inhibiting m6A methylation of PLIN2. The findings suggest that targeting the METTL3‐PLIN2 axis has potential therapeutic benefits for IPF.

## Author Contributions

**Xianzhi Du:** writing – review and editing, supervision, resources. **Qiping Liu:** writing – original draft, writing – review and editing. **Rui Xu:** writing – review and editing, software, formal analysis.

## Funding

The study was supported by General Program of Chongqing Natural Science Foundation (Number: cstc2021jcyj‐msxmX0216, Funding Agency: Chongqing Municipal Science and Technology Bureau), Joint General Program of Chongqing Science and Health Research (Number: 2022MSXM144, Funding Agency: Chongqing Municipal Science and Technology Bureau and Chongqing Municipal Health Commission), Future Medicine Youth Innovation Team Project of Chongqing Medical University (Number: W0118, Funding Agency: Chongqing Medical University).

## Ethics Statement

This study was approved by the Ethics Committee of Second Affiliated Hospital of Chongqing Medical University. All animal experiments should comply with the ARRIVE guidelines. All methods were carried out in accordance with relevant guidelines and regulations.

## Consent

The authors have nothing to report.

## Conflicts of Interest

The authors declare no conflicts of interest.

## Data Availability

The datasets used and/or analysed during the current study are available from the corresponding author on reasonable request.

## References

[1] P. Spagnolo , J. A. Kropski , M. G. Jones , et al., “Idiopathic Pulmonary Fibrosis Disease Mechanisms and Drug Development,” Pharmacology & Therapeutics 222 (2021): 107798. 33359599 10.1016/j.pharmthera.2020.107798 PMC8142468

[2] Q. Mei , Z. Liu , H. Zuo , Z. Yang , and J. Qu , “Idiopathic Pulmonary Fibrosis An Update on Pathogenesis,” Frontiers in Pharmacology 12 (2021): 797292. 35126134 10.3389/fphar.2021.797292 PMC8807692

[3] D. Leng , X. Huang , J. Yi , H. Zhao , and Y. Zhang , “HYAL1 Is Downregulated in Idiopathic Pulmonary Fibrosis and Inhibits HFL‐1 Fibroblast Proliferation When Upregulated,” BioMed Research International 2020 (2020): 3659451. 32258117 10.1155/2020/3659451 PMC7086424

[4] A. Koudelka , V. Cechova , M. Rojas , et al., “Fatty Acid Nitroalkene Reversal of Established Lung Fibrosis,” Redox Biology 50 (2022): 102226. 35150970 10.1016/j.redox.2021.102226 PMC8844680

[5] T. Koudstaal and M. S. Wijsenbeek , “Idiopathic Pulmonary Fibrosis,” Presse Médicale 52 (2023): 104166. 37156412 10.1016/j.lpm.2023.104166

[6] A. J. Podolanczuk , C. C. Thomson , M. Remy‐Jardin , et al., “Idiopathic Pulmonary Fibrosis State of the Art for 2023,” European Respiratory Journal 61 (2023): 2200957. 36702498 10.1183/13993003.00957-2022

[7] B. J. Moss , S. W. Ryter , and I. O. Rosas , “Pathogenic Mechanisms Underlying Idiopathic Pulmonary Fibrosis,” Annual Review of Pathology 17 (2022): 515–546. 10.1146/annurev-pathol-042320-030240 34813355

[8] R. Chen and J. Dai , “Lipid Metabolism in Idiopathic Pulmonary Fibrosis From Pathogenesis to Therapy,” Journal of Molecular Medicine (Berlin, Germany) 101 (2023): 905–915. 37289208 10.1007/s00109-023-02336-1

[9] Y. Tian , C. Duan , J. Feng , J. Liao , Y. Yang , and W. Sun , “Roles of Lipid Metabolism and Its Regulatory Mechanism in Idiopathic Pulmonary Fibrosis A Review,” International Journal of Biochemistry & Cell Biology 155 (2023): 106361. 36592687 10.1016/j.biocel.2022.106361

[10] R. Karthiya and P. Khandelia , “m6A RNA Methylation Ramifications for Gene Expression and Human Health,” Molecular Biotechnology 62 (2020): 467–484. 32840728 10.1007/s12033-020-00269-5

[11] Y. An and H. Duan , “The Role of m6A RNA Methylation in Cancer Metabolism,” Molecular Cancer 21 (2022): 14. 35022030 10.1186/s12943-022-01500-4 PMC8753874

[12] L. Li , N. Xu , J. Liu , Z. Chen , X. Liu , and J. Wang , “m6A Methylation in Cardiovascular Diseases From Mechanisms to Therapeutic Potential,” Frontiers in Genetics 13 (2022): 908976. 35836571 10.3389/fgene.2022.908976 PMC9274458

[13] X. Dai , Y. Cheng , W. Luo , et al., “m6A Ribonucleic Acid Methylation in Fibrotic Diseases of Visceral Organs,” Small Science 5 (2024): 2400308. 40213062 10.1002/smsc.202400308 PMC11934900

[14] W. Ye , X. Lv , S. Gao , Y. Li , J. Luan , and S. Wang , “Emerging Role of m6A Modification in Fibrotic Diseases and Its Potential Therapeutic Effect,” Biochemical Pharmacology 218 (2023): 115873. 37884198 10.1016/j.bcp.2023.115873

[15] G. Huang , S. Huang , and H. Cui , “Effect of M6A Regulators on Diagnosis, Subtype Classification, Prognosis and Novel Therapeutic Target Development of Idiopathic Pulmonary Fibrosis,” Frontiers in Pharmacology 13 (2022): 993567. 36518679 10.3389/fphar.2022.993567 PMC9742476

[16] J. Zhang , Y. Zhang , Z. Wang , et al., “Genes Related to N6‐Methyladenosine in the Diagnosis and Prognosis of Idiopathic Pulmonary Fibrosis,” Frontiers in Genetics 13 (2022): 1102422. 36685949 10.3389/fgene.2022.1102422 PMC9846232

[17] E. Sendinc and Y. Shi , “RNA m6A Methylation Across the Transcriptome,” Molecular Cell 83 (2023): 428–441. 36736310 10.1016/j.molcel.2023.01.006

[18] J. X. Zhang , P. J. Huang , D. P. Wang , et al., “M(6)A Modification Regulates Lung Fibroblast‐To‐Myofibroblast Transition Through Modulating KCNH6 mRNA Translation,” Molecular Therapy 29 (2021): 3436–3448. 34111558 10.1016/j.ymthe.2021.06.008 PMC8636177

[19] C. Sztalryd and D. L. Brasaemle , “The Perilipin Family of Lipid Droplet Proteins Gatekeepers of Intracellular Lipolysis,” Biochimica et Biophysica Acta ‐ Molecular and Cell Biology of Lipids 1862 (2017): 1221–1232. 28754637 10.1016/j.bbalip.2017.07.009 PMC5595658

[20] M. Russo , R. A. Montone , D. D'Amario , et al., “Role of Perilipin 2 in Microvascular Obstruction in Patients With ST‐Elevation Myocardial Infarction,” European Heart Journal Acute Cardiovascular Care 10 (2021): 633–642. 33620432 10.1093/ehjacc/zuaa004

[21] D. J. Orlicky , A. E. Libby , E. S. Bales , et al., “Perilipin‐2 Promotes Obesity and Progressive Fatty Liver Disease in Mice Through Mechanistically Distinct Hepatocyte and Extra‐Hepatocyte Actions,” Journal of Physiology 597 (2019): 1565–1584. 30536914 10.1113/JP277140 PMC6418763

[22] Y. Zhang , J. Fu , C. Li , et al., “Omentin‐1 Induces Mechanically Activated Fibroblasts Lipogenic Differentiation Through pkm2/Yap/Ppargamma Pathway to Promote Lung Fibrosis Resolution,” Cellular and Molecular Life Sciences 80 (2023): 308. 37768341 10.1007/s00018-023-04961-y PMC11072733

[23] N. Fillmore , V. Hou , J. Sun , D. Springer , and E. Murphy , “Cardiac Specific Knock‐Down of Peroxisome Proliferator Activated Receptor Alpha Prevents Fasting‐Induced Cardiac Lipid Accumulation and Reduces Perilipin 2,” PLoS One 17 (2022): e0265007. 35259201 10.1371/journal.pone.0265007 PMC8903264

[24] X. Shi , Y. Chen , M. Shi , et al., “The Novel Molecular Mechanism of Pulmonary Fibrosis Insight Into Lipid Metabolism From Reanalysis of Single‐Cell RNA‐Seq Databases,” Lipids in Health and Disease 23 (2024): 98. 38570797 10.1186/s12944-024-02062-8 PMC10988923

[25] P. Wang , D. Xie , T. Xiao , et al., “H3K18 Lactylation Promotes the Progression of Arsenite‐Related Idiopathic Pulmonary Fibrosis Via YTHDF1/m6A/NREP,” Journal of Hazardous Materials 461 (2024): 132582. 37742376 10.1016/j.jhazmat.2023.132582

[26] C. Zhang , L. Chen , C. Xie , et al., “YTHDC1 Delays Cellular Senescence and Pulmonary Fibrosis by Activating ATR in an m6A‐Independent Manner,” EMBO Journal 43 (2024): 61–86. 38177310 10.1038/s44318-023-00003-2 PMC10883269

[27] B. Tu , K. Song , Y. Zhou , et al., “METTL3 Boosts Mitochondrial Fission and Induces Cardiac Fibrosis by Enhancing LncRNA GAS5 Methylation,” Pharmacological Research 194 (2023): 106840. 37379961 10.1016/j.phrs.2023.106840

[28] H. R. Jung , J. Lee , S. P. Hong , et al., “Targeting the m(6)A RNA Methyltransferase METTL3 Attenuates the Development of Kidney Fibrosis,” Experimental & Molecular Medicine 56 (2024): 355–369. 38297163 10.1038/s12276-024-01159-5 PMC10907702

[29] B. Shu , Y. X. Zhou , H. Li , R. Z. Zhang , C. He , and X. Yang , “The METTL3/MALAT1/PTBP1/USP8/TAK1 Axis Promotes Pyroptosis and M1 Polarization of Macrophages and Contributes to Liver Fibrosis,” Cell Death Discovery 7 (2021): 368. 34839365 10.1038/s41420-021-00756-x PMC8627510

[30] V. Suryadevara , R. Ramchandran , D. W. Kamp , and V. Natarajan , “Lipid Mediators Regulate Pulmonary Fibrosis Potential Mechanisms and Signaling Pathways,” International Journal of Molecular Sciences 21 (2020): 4257. 32549377 10.3390/ijms21124257 PMC7352853

[31] Y. Nakamura and Y. Shimizu , “Cellular and Molecular Control of Lipid Metabolism in Idiopathic Pulmonary Fibrosis Clinical Application of the Lysophosphatidic Acid Pathway,” Cells 12 (2023): 12. 10.3390/cells12040548 PMC9954511 36831215

[32] M. Conte , C. Franceschi , M. Sandri , and S. Salvioli , “Perilipin 2 and Age‐Related Metabolic Diseases A New Perspective,” Trends in Endocrinology and Metabolism 27 (2016): 893–903. 27659144 10.1016/j.tem.2016.09.001

[33] D. den Braanker , R. Maas , G. van Mierlo , et al., “Primary Focal Segmental Glomerulosclerosis Plasmas Increase Lipid Droplet Formation and Perilipin‐2 Expression in Human Podocytes,” International Journal of Molecular Sciences 24 (2022): 194. 36613637 10.3390/ijms24010194 PMC9820489

[34] J. E. Norman , H. H. Aung , D. W. Wilson , and J. C. Rutledge , “Inhibition of Perilipin 2 Expression Reduces Pro‐Inflammatory Gene Expression and Increases Lipid Droplet Size,” Food & Function 9 (2018): 6245–6256. 30402637 10.1039/c8fo01420e PMC6292725

[35] M. Loix , E. Wouters , S. Vanherle , et al., “Perilipin‐2 Limits Remyelination by Preventing Lipid Droplet Degradation,” Cellular and Molecular Life Sciences 79 (2022): 515. 36100764 10.1007/s00018-022-04547-0 PMC11803036

[36] Y. Zhou , Y. Wang , C. Ni , et al., “Perilipin 2 Protects Against Lipotoxicity‐Induced Islet Fibrosis by Inducing Islet Stellate Cell Activation Phenotype Changes,” BioMed Research International 2022 (2022): 4581405. 35845956 10.1155/2022/4581405 PMC9279040

[37] C. P. Najt , S. Senthivinayagam , M. B. Aljazi , et al., “Liver‐Specific Loss of Perilipin 2 Alleviates Diet‐Induced Hepatic Steatosis, Inflammation, and Fibrosis,” American Journal of Physiology. Gastrointestinal and Liver Physiology 310 (2016): G726–G738. 26968211 10.1152/ajpgi.00436.2015 PMC4867327

[38] Y. Lu , Z. Liu , Y. Zhang , et al., “METTL3‐Mediated m6A RNA Methylation Induces the Differentiation of Lung Resident Mesenchymal Stem Cells Into Myofibroblasts Via the miR‐21/PTEN Pathway,” Respiratory Research 24 (2023): 300. 38017523 10.1186/s12931-023-02606-z PMC10683095

[39] F. Qi , L. Liu , C. Peng , et al., “m6A Methylation‐Modified hsa_circ_0044226 Attenuates Pulmonary Fibrosis by Suppressing Ferroptosis,” Pulmonary Circulation 15 (2025): e70197. 41179914 10.1002/pul2.70197 PMC12576025

[40] A. Chiariello , L. Rossetti , S. Valente , et al., “Downregulation of PLIN2 in Human Dermal Fibroblasts Impairs Mitochondrial Function in an Age‐Dependent Fashion and Induces Cell Senescence via GDF15,” Aging Cell 23 (2024): e14111. 38650174 10.1111/acel.14111 PMC11113257

[41] E. Vásquez‐Pacheco , M. Marega , A. Lingampally , et al., “Highlighting Fibroblast Plasticity in Lung Fibrosis The WI‐38 Cell Line as a Model for Investigating the Myofibroblast and Lipofibroblast Switch,” Theranostics 14 (2024): 3603–3622. 38948058 10.7150/thno.93519 PMC11209726

